# Effects of *Alpinia oxyphylla* stems and leaves extracts on immune function, antioxidant function, and microbial flora composition in the intestinal tract of Jiaji ducks

**DOI:** 10.3389/fvets.2025.1662049

**Published:** 2025-11-27

**Authors:** Yongliang Wang, Zhe Chen, Weiqi Peng, Chengjun Hu, Hongzhi Wu, Renlong Lyu, Bie Tan, Tieshan Xu, Fengjie Ji

**Affiliations:** 1 Tropical Crops Genetic Resources Research Institute, Chinese Academy of Tropical Agricultural Sciences, Haikou, China; 2 Animal Nutritional Genome and Germplasm Innovation Research Center, College of Animal Science and Technology, Hunan Agricultural University, Changsha, Hunan, China

**Keywords:** *Alpinia oxyphylla*, Jiaji duck, intestinal immunity, intestinal antioxidation, microbiota

## Abstract

*Alpiniae Oxyphyllae* (*A. oxyphylla*) has been widely used as a traditional herbal medicine, and our previous studies have shown that its extracts from stems and leaves have a beneficial role in Jiaji ducks health. This study aimed to further investigate the effects of the *Alpinia oxyphylla* stems and leaves extracts (AOE) on the intestinal health of fattening Jiaji ducks. Four hundred and eighty 42-day-old Jiaji ducks were randomly allocated and equally divided into four treatment groups. The control group (CK) was fed a basal diet, and the AOE groups (groups Y1, Y2 and Y3) were fed with supplement 30, 80, or 130 mg/kg of AOE diets for 49 days. The results showed that the stems and leaves of *A. oxyphylla* contain abundant anti-inflammatory and antioxidant compounds, such as fatty acyls, terpenoids, flavonoids, and phenols. Compared with the CK group, the AOE groups (Y1, Y2, and Y3) exhibited significant effects in the immune and antioxidant functions. Among them, the mRNA expression of interleukin-10 (*IL-10*), heme oxidase-1 (*HO-1*) and nuclear factor E2-related factor 2 (*Nrf2*) in jejunal mucosa was significantly up-regulated (*p* = 0.005, *p* = 0.007, *p* = 0.005) in the AOE groups (Y1, Y2, and Y3). The levels of lysozyme, intestinal alkaline phosphatase (AKP), glutathione peroxidase (GSH-Px) and anti-superoxide anion radical (ASA) were markedly increased (*p* < 0.05) in jejunal mucosa of the Y1 groups. Moreover, the Spearman analysis demonstrated that the microbial biomarkers *Ruminiclostridium_9* were positively correlated (*p* < 0.05) with *HO-1* and *Nrf2* in jejunal mucosa, and *Bacteroidales* were positively correlated (*p* < 0.05) with lysozyme activity. In contrast, *Lachnospiraceae_unclassified* and *Firmicutes_unclassified* were negatively correlated (*p* < 0.05) with lysozyme activity. In conclusion, dietary supplementation with 30 mg/kg of AOE is the most suitable dose to maintain the healthy homeostasis of the intestinal tract of Jiaji ducks, which can inhibit intestinal inflammatory responses, improve antioxidant function and alter the composition of intestinal microorganisms.

## 1 Introduction

Jiaji Duck, a high-quality Muscovy duck breed native to Hainan Province (China), has been bred for over two centuries. It is known for its large breast, thin skin, soft bone, tender meat, low cholesterol, high fat with relatively low grease, and high nutritional value, which makes it one of Hainan’s “Four Famous Culinary Specialties” and is widely consumed (1, 2). However, the high-density breeding of Jiaji ducks has given rise to significant health and disease concerns, impeding the industry’s healthy development (3). *Alpinia oxyphylla* (*A. oxyphylla*), a plant belonging to the ginger family, is primarily produced in Hainan Province (China). The mature fruit of *A. oxyphylla* is regarded as one of China’s “Four Great Southern Medicines,” known for its anti-diarrhea, anti-inflammatory, antioxidant, and neuroprotective properties (4–6). However, the *A. oxyphylla* stems and leaves are discarded, which result in resource wastage. In recent studies, Liu et al. (7) reported that the supplementation of *A. oxyphyllae* fructus powder could improve production performance and egg quality of hens by modulating reproductive hormones, antioxidant capacity, immunity, intestinal barrier, and cecal microbiota. Numerous chemical constituents beneficial to health have been isolated from *A. oxyphylla* stems and leaves, including flavonoids, diarylheptanoids, sesquiterpenes, sterols, and their glycosides (8). These functional constituents have multiple bioactivities, such as antioxidant, neuroprotective, anticancer, anti-inflammatory, antidiabetic, and antiosteoporotic activities (9, 10). The results of our previous studies found that the addition of the *Alpinia oxyphylla* stems and leaves extracts (AOE) to the diets of Jiaji ducks, which could significantly improve their health status by decreasing blood lipid metabolism, enhancing meat quality, maintaining intestinal structural integrity, and modulating the composition of intestinal microorganisms (11). These findings suggest that AOE may have medicinal value similar to *A. oxyphylla* fruit, but further analysis is needed to clarify the active components in AOE.

The intestine is the main organ responsible for immune defense and nutrient absorption, so that maintaining intestinal health is imperative to protect Jiaji ducks (12, 13). Our previous studies demonstrated that adding AOE significantly increased jejunal villus height and villus height to crypt depth ratio in fattening Jiaji ducks. Furthermore, a significant effect was observed on the abundance of *Bacteroidales_unclassified* and *Ruminococcaceae_unclassified* at the microbial genus level (11). Currently, fewer studies are addressing the effects of the *Alpinia oxyphylla* stems and leaves on the intestinal health of meat ducks. However, curcumin, a plant extracts from the same ginger family as AOE, has been widely researched. It has been demonstrated to possess the capacity to effectively maintain intestinal health in meat ducks by modulating the enzyme activities of intestinal antioxidants and immunity, enhancing the intestinal barrier, and regulating the gene expression of inflammation and antioxidants (14). However, the effects of AOE on immune function and antioxidant function in the intestinal tract of Jiaji ducks remain to be elucidated, and further studies on the regulatory effects of AOE on their intestinal health are required. Consequently, the main chemical components of the *Alpinia oxyphylla* stems and leaves were analyzed by ultra-high performance liquid chromatography-tandem time-of-flight mass spectrometry (UHPLC-TOF-MS). The AOE was then prepared, and the effect of AOE on maintaining intestinal health was further evaluated by using Jiaji ducks at the fattening stage as the study subjects.

## 2 Materials and methods

### 2.1 Plant material and preparation of extracts

The stems and leaves of *A. oxyphylla* were gathered from the Tropical Botanical Garden (Danzhou, China) and identified by Professor Wang Maoyuan. The AOE was prepared using the method of Ji et al. (11). In brief, the plant tissues were air-dried at 60 °C, and ground to powder with a stainless-steel blender. The powder was extracted three times in a reflux condenser for 1.5 h each with 95% ethanol at 55/60 °C. The solution was combined and filtered. Solvents were removed by using a rotary vacuum evaporator. Finally, the crude extracts was condensed in a freezer-dryer, and brown powder was obtained.

### 2.2 Experimental design, animals, and diets

A total of 480 42-day-old Healthy Jiaji ducks (initial body weight 1675.8 ± 44.2 g, male:female = 1:1) were purchased from Hainan Chuanwei Duck Breeding Co. Ltd. (Qionghai, China) and subsequently reared in the demonstration farm of the company. The 480 Jiaji ducks were randomly allocated to four groups equally (6 replicates per group, 20 ducks per replicate). The different treatment groups included: (1) CK group (basal diet without AOE); (2) Y1 group (basal diet + 30 mg/kg AOE); (3) Y2 group (basal diet + 80 mg/kg AOE); (4) Y3 group (basal diet + 130 mg/kg AOE). The duck pens were equipped with feeders, drinkers, and non-slip plastic mesh flooring. Each pen was provided with a 20-W incandescent white fluorescent lamp, and 24-h continuous incandescent lighting was provided. The temperature was set initially at 30 °C and reduced gradually to 20 °C at the end of the experiment. The relative humidity in duck house was 65–75%. Daily feed intake, body weight, and health status were recorded for a trial period of 49 days. The basal diet formulations were presented in Supplementary Table S1, and the growth performance was shown in the previous paper by Ji et al. (11).

### 2.3 Sample collection and treatment

After the feeding trial, two ducks with comparable mean weights from each replicate were slaughtered on the same day. A segment measuring 10 cm was taken from the proximal segment of the small intestine and gently rinsed with 0.9% normal saline. Subsequently, mucosal tissue was scraped using sterile slides and wrapped in aluminum foil, and immediately stored in liquid nitrogen. For homogenate preparation, the jejunal mucosa was transferred to a sterile mortar and ground into powder under continuous liquid nitrogen cooling. The jejunal mucosa powder was accurately weighed (50 ± 5 mg) and transferred to tubes containing DNase/RNase-Free, prepared at a 1∶9 ratio (mg∶μL) with pre-cooled sterile saline. Thereafter, the solution was then homogenized (0 °C) and centrifuged (10,000 rpm, 4 °C, 10 min). The resulting supernatant was collected and stored at −80 °C for subsequent analysis. The cecal contents were collected into 10 mL sterile tubes and stored at −80 °C for further analysis.

### 2.4 Indicator tests

#### 2.4.1 Analysis of chemical components in *A. oxyphylla* stems and leaves

In order to ascertain the chemical composition of *A. oxyphylla* stems and leaves, the fresh samples (50 ± 5 mg) were weighed and thoroughly mixed in 1 mL pre-cooled aqueous solution (methanol∶acetonitrile∶water = 2∶2∶1, v∶v∶v). The mixture was homogenized for 30 s, then subjected to ultrasonic extraction (0 °C, 40 kHz, 30 min) and centrifugation (4 °C, 14,000 rpm, 10 min). The supernatant was collected, concentrated under reduced pressure, and lyophilized. Subsequently, the dried residue was redissolved in 100 μL acetonitrile water (1∶1, v∶v), followed by vortex (30 s), and centrifugation (4 °C, 14,000 rpm, 15 min) again. This step was repeated once to enhance purity. The final supernatant was filtered through a 0.22-μm membrane for further analysis.

The samples were analyzed using UHPLC-TOF-MS (Agilent 1290 Infinity LC, Agilent Technologies, United States) by the method of de Macedo et al. (15). Chromatographic separation was performed on an Agilent Poroshell 120 EC-C18 column (2.1 × 100 mm, 1.9 μm) maintained at 40 °C. The mobile phase consisted of (A) water containing 25 mM ammonium acetate and 0.5% formic acid; (B) acetonitrile (or methanol) containing 0.1% formic acid. A gradient elution program was applied: 0–0.5 min (5% B), 0.5–10 min (5–100%), 10.0–12.0 min (100% B), 12.0–12.1 min (100–5% B), 12.1–16 min (5% B), at a flow rate of 0.4 mL/min with a 2-μL injection volume. Mass spectrometry parameters included electrospray ionization (ESI) in positive/negative switching mode, nebulizer pressure 20 psi, drying temperature 300 °C, capillary voltage ± 5.5 kV, and the collision energy 35 (15 eV spread). Metabolites were identified by matching retention times, exact mass (within 25 ppm), and MS/MS fragmentation patterns against the Zhongke New Life reference database (Shanghai Applied Protein Technology).

#### 2.4.2 Enzyme-linked immunosorbent assay

The concentrations of lysozyme, alkaline phosphatase (AKP), secretory immunoglobulin A (sIgA), and mucin 2 (MUC2) in the jejunal mucosa were measured using enzyme-linked immunosorbent assays (Shanghai Kexing Trading Co., Shanghai, China).

#### 2.4.3 Digestive enzyme and antioxidant enzyme activity assay

Protein in the jejunal mucosa supernatant were quantified using the BCA protein concentration kit (Shanghai Biyuntian Biotechnology Co., Ltd. Shanghai, China). Then the activities and levels of lactase, sucrase, maltase, superoxide dismutase (SOD), malondialdehyde (MDA), glutathione peroxidase (GSH-Px), catalase (CAT), anti-hydroxyl radical (AHR), and anti-superoxide anion (ASA) were tested by the kit (Nanjing Jianjian Bioengineering Research Institute, Nanjing, China).

#### 2.4.4 Determination of mRNA expression of jejunal mucosa genes

RNA extraction, primer design, reverse transcription, real-time quantitative PCR (RT-qPCR) reaction conditions, and the calculation of relative quantification (2^−ΔΔCt^ method) were performed as previously described by Li et al. (16). The sequences of all primers are listed in Table 1 and *β-Actin* was used as an internal reference gene.

**Table 1:** Primer sequences used in RT-qPCR.

Genes	Primer (5′-3′)	Length (bp)
*β-actin*	F: GCTATGTCGCCCTGGATTT	160
	R: GGATGCCACAGGACTCCATAC	
*IFN-γ*	F: GACTTGCCAGACCTACTGCTTG	251
	R: TCTTTAGCATTTCCAAGTACAGGGT	
*TGF-β*	F: ACAGAGCCGCCACTTGTGAA	160
	R: TCCATCGCCGAGACATCAAAT	
*TNF-α*	F: ACAGGACAGCCTATGCCAACAA	118
	R: TCTGATTACAGGAAGGGCAACA	
*IL-1β*	F: CTGGGCATCAAGGGCTACAA	131
	R: CGGTAGAAGATGAAGCGGGT	
*IL-6*	F: AGCACAAAGCATCTGGCAAC	192
	R: AACAGCCCTCACGGTTTTCT	
*IL-10*	F: CTGCCTCCACTTGTCTGACCT	95
	R: CATCGTCTTGGGATTGAAAGTAGTC	
*Claudin-1*	F: GTGTATTTGTTGCCGTGACTGG	155
	R: AGCCACTCTGTTGCCATACCAT	
*Occludin*	F: GTGATTACGGCTACGACTACGG	196
	R: AAACCGTAGCCGTAGTCCCAG	
*ZO-1*	F: CTGTGGGTAACTCCATCTTCCG	196
	R: CCCTGTGAAGAGTCACTGTGTGTT	
*CAT*	F: AGGGTAACTGGGATCTTGTGGG	159
	R: AAGACTCAGGGCGAAGACTCCA	
*HIF1α*	F: TTCAGGCAGTTGGAATTGGGT	199
	R: AGTTGGGGTAGTCCACTTTCATC	
*HO-1*	F: GATGGACCTTGCTACTAAGAAACGA	223
	R: TGTCTGACTCCTGCTTGTCCTCT	
*Nrf1*	F: CGACGGGTAAGAAACGAAAGC	253
	R: GCAGACTCCAGGTCTTCCAGAAT	
*Nrf2*	F: GCTGGAGTTAGACGAGGAGACA	111
	R: GAAGTATGCGTGCTCTGTGAAA	
*PGC-1α*	F: ACAGCAATGAACCCGCCAATA	117
	R: AAGGCAATCCATCCTCATCCAC	
*GPX1*	F: GAGAATGCCACCAACGAGGAG	212
	R: ACGGGCGACCAGATGATGTA	
*SOD1*	F: TAAAGGAGGAGTAGCAGATGTGGA	196
	R: TCAGCACTTGGCTATTCCGATG	

^*^β-actin, beta-actin; IFN-γ, interferon gamma; TGF-β, transforming growth factor-beta; TNF-α, tumor necrosis factor-alpha; IL-1β, interleukin-1 beta; IL-6, interleukin-6; IL-10, interleukin-10; ZO-1, zonula occludens protein-1; CAT, catalase; HIF1α, hypoxia inducible factor 1 subunit alpha; HO-1, heme oxygenase 1; Nrf1, nuclear factor erythroid 1-related factor 1; Nrf2, nuclear factor erythroid 2-related factor 2; PGC-1α, peroxisome proliferator-activated receptor gamma (PPAR-G) coactivator 1 alpha; GPX1, glutathione peroxidase 1; SOD1, superoxide dismutase 1; F, forward; R, reverse.

#### 2.4.5 Spearman correlation analysis of cecal microbiota

DNA extraction, PCR amplification, 16S rDNA data analysis, and differential analysis of microbial genus in cecum contents were shown in the previous paper by Ji et al. (11). In the present study, spearman correlation was used to further evaluate the relationship between the indices of oxidation and immunity in jejunal mucosa with the abundance of microbial genus levels in cecum contents.

#### 2.4.6 Determination of short-chain fatty acid content in cecal contents

The short-chain fatty acids (SCFA) content was determined using the methodology outlined by Liu et al. (17). Briefly, cecal contents were weighed (1 ± 0.05 g), transferred to a sterile tube, and mixed with 1 mL of pre-cooled ultrapure water. The mixture was homogenized (30 s), followed by shaking and centrifugation (4 °C, 15,000 rpm, 15 min) to collect the supernatant, which was then mixed with 25% metaphosphoric acid at a 1∶9 ratio (v∶v, supernatant∶metaphosphoric acid) and incubated (4 °C, 30 min). The final solution was filtered through a 0.22-μm membrane, which was then analyzed by gas chromatography (5975C, Agilent Technologies, United States).

### 2.5 Statistical analysis

The results were presented as means with respective standard error of the mean (SEM). The data were checked for normality and homogeneity before analysis with the Shapiro–Wilk and Levene tests, respectively. The data were analyzed by one-way ANOVA procedures of SPSS v. 22.0 software (SPSS Inc., Chicago, IL, United States), followed by Duncan’s multiple comparison tests. Spearman’s correlation coefficient was used to assess the relationships between the environmental factors and the relative abundances of microbial genera. Significant differences between means were indicated by *p* < 0.05.

## 3 Results

### 3.1 Chemical components of *Alpinia oxyphylla* stems and leaves

**Figure 1:**
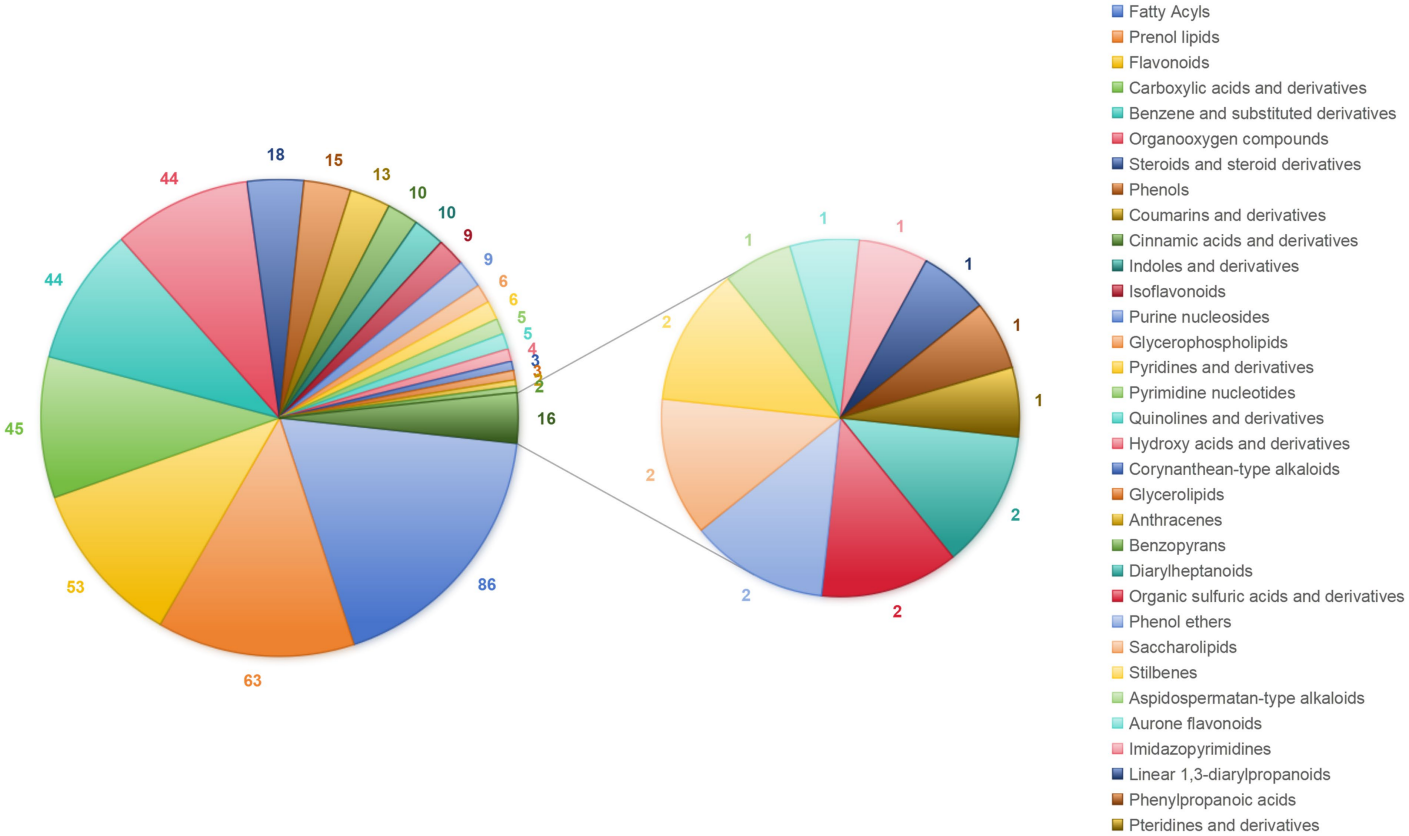
Chemical components of *A. oxyphylla* stems and leaves. Combining the positive and negative ion modes.

After detection and identification, a total of 696 metabolites were identified by combining positive and negative ion modes, with 421 compounds in the positive ion mode and 275 in the negative ion mode. The metabolites were classified according to their chemical taxonomy, and 471 metabolites were annotated in the both modes (Figure 1). Among them, the top five are ranked as follows: 86 fatty acyls, 63 isoprene lipids, 53 flavonoids, 45 carboxylic acids and their derivatives, and 44 benzene and its derivatives.

### 3.2 Effect of AOE on jejunal immune function in ducks

**Figure 2:**
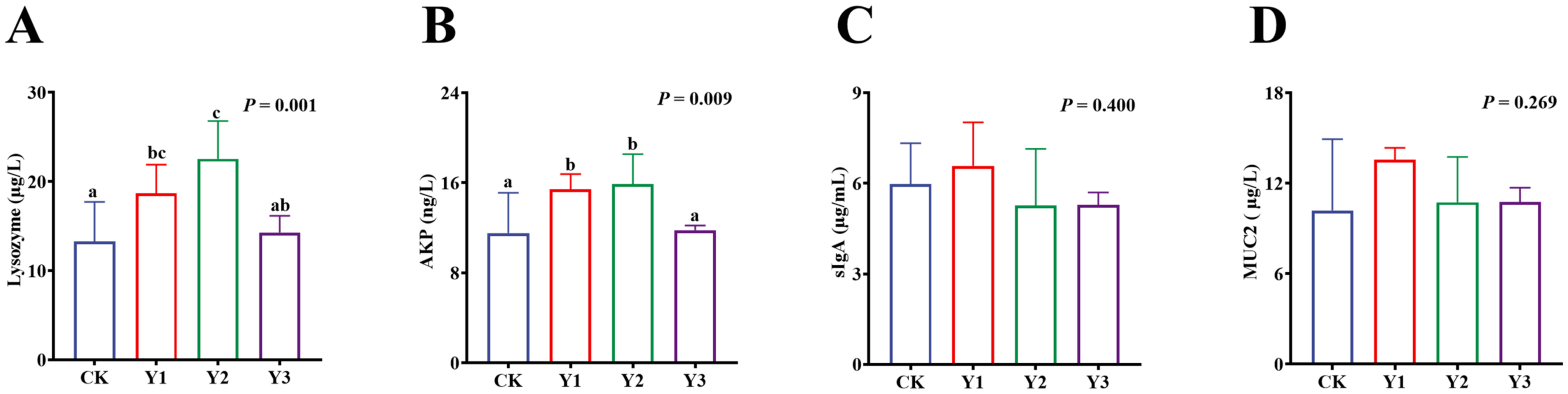
Effect of AOE on the immune enzyme in the jejunal mucosa of ducks. AKP: Alkaline phosphatase; sIgA: Secretory immunoglobulin A; MUC2: Mucin. ^a,b^ Bars with different superscripts indicate a significant difference (*n* = 6, *p* < 0.05).

**Figure 3:**
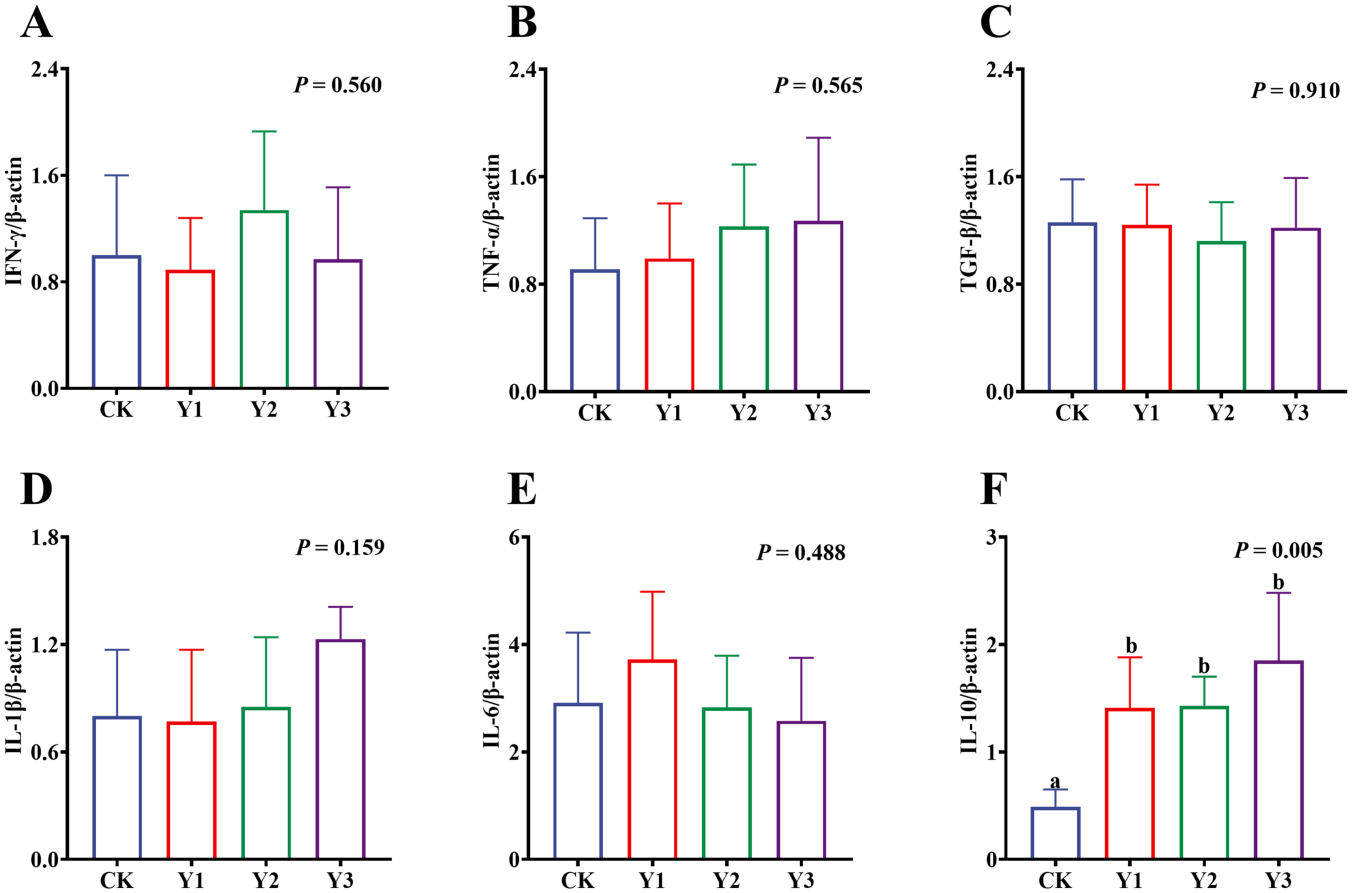
Effect of AOE on mRNA relative expression of immune-related genes in the jejunal mucosa of ducks. β-actin: beta-actin; IFN-γ: interferon gamma; TNF-α: tumor necrosis factor-alpha; TGF-β: transforming growth factor-beta; IL-1β: interleukin-1 beta; IL-6: interleukin-6; IL-10: interleukin-10. ^a,b^ Bars with different superscripts indicate a significant difference (*n* = 6, *p* < 0.05).

**Figure 4:**
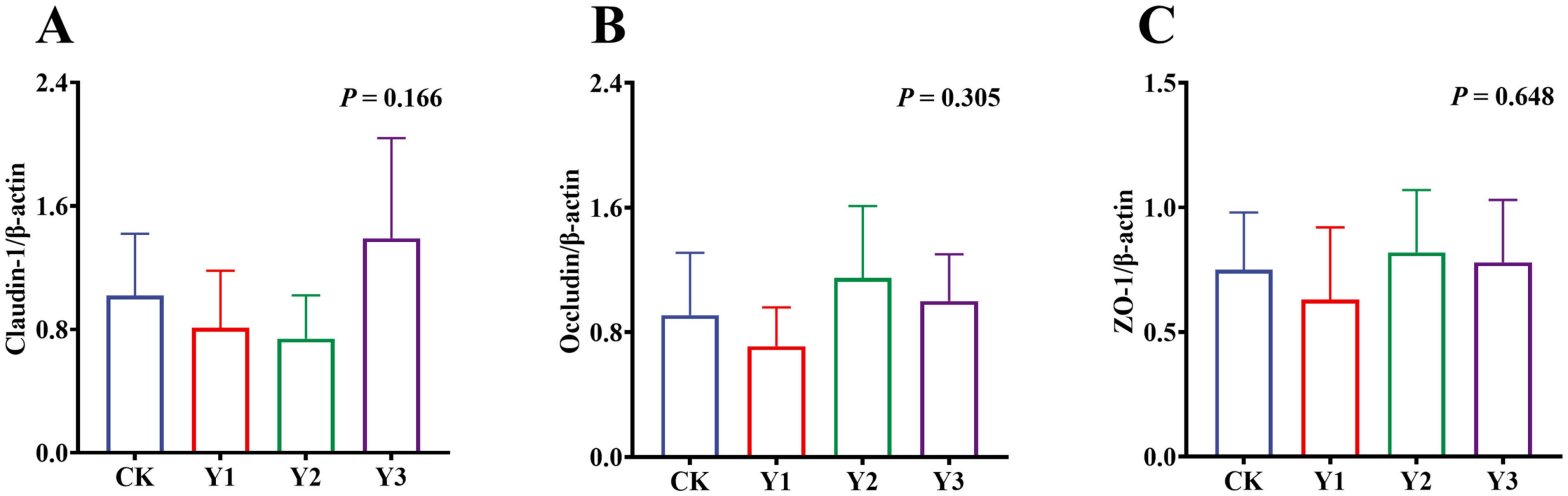
Effect of AOE on mRNA relative expression of tight junction protein-related genes in the jejunal mucosa of ducks. β-actin: beta-actin; ZO-1: Zonula Occludens-1. ^a,b^ Bars with different superscripts indicate a significant difference (*n* = 6, *p* < 0.05).

Compared to the CK group, the lysozyme and AKP activity in the Y1 and Y2 groups were significantly (*p* < 0.05) increased (Figures 2A,B). In contrast, the sIgA and MUC2 concentrations showed no significant (*p* > 0.05) differences between the groups (Figures 2C,D). The RT-qPCR analysis revealed that the *IL-10* mRNA expression in the AOE groups (Y1, Y2 and Y3) were significantly (*p* = 0.005) up-regulated (Figure 3F). However, the mRNA expression levels of the *IFN-γ*, *TNF-α*, *TGF-β*, *IL-1β*, and *IL-6* in these groups remained unchanged (*p* > 0.05) (Figures 3A–E). Moreover, there were also no significant (*p* > 0.05) differences in the mRNA expression levels of *Claudin-1*, *Occludin*, and *ZO-1* (Figures 4A–C).

### 3.3 Effect of AOE on digestive enzyme and antioxidant function of the jejunal mucosa in ducks

**Figure 5:**
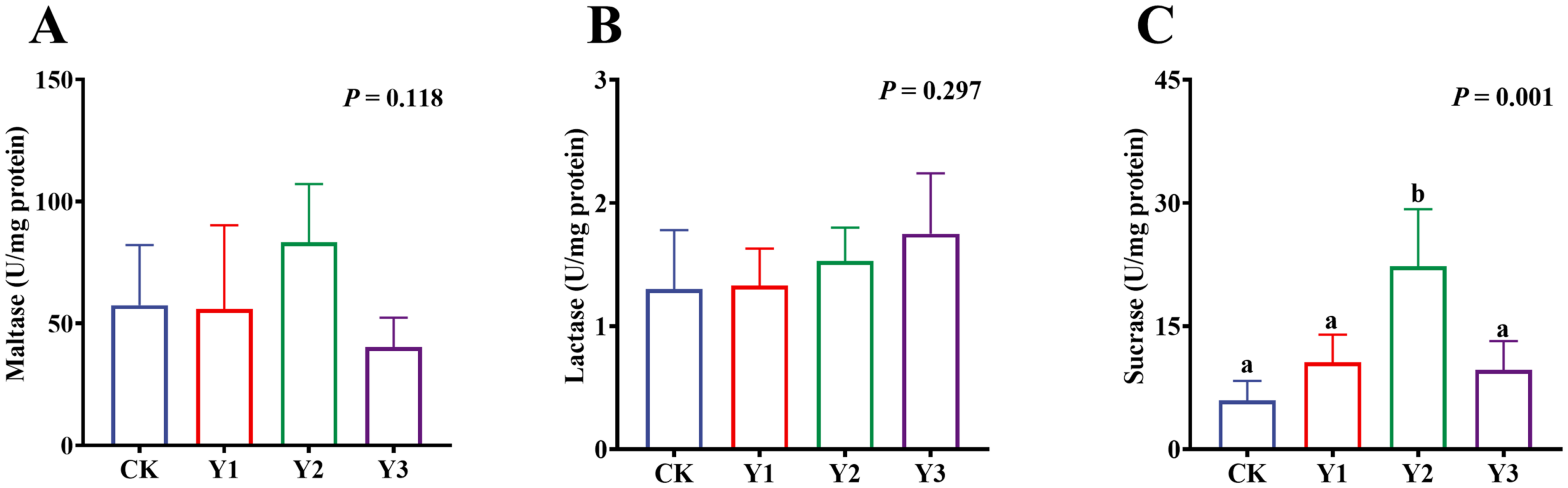
Effect of AOE on the digestive enzymes in the jejunal mucosa of ducks. ^a,b^ Bars with different superscripts indicate a significant difference (*n* = 6, *p* < 0.05).

**Figure 6:**
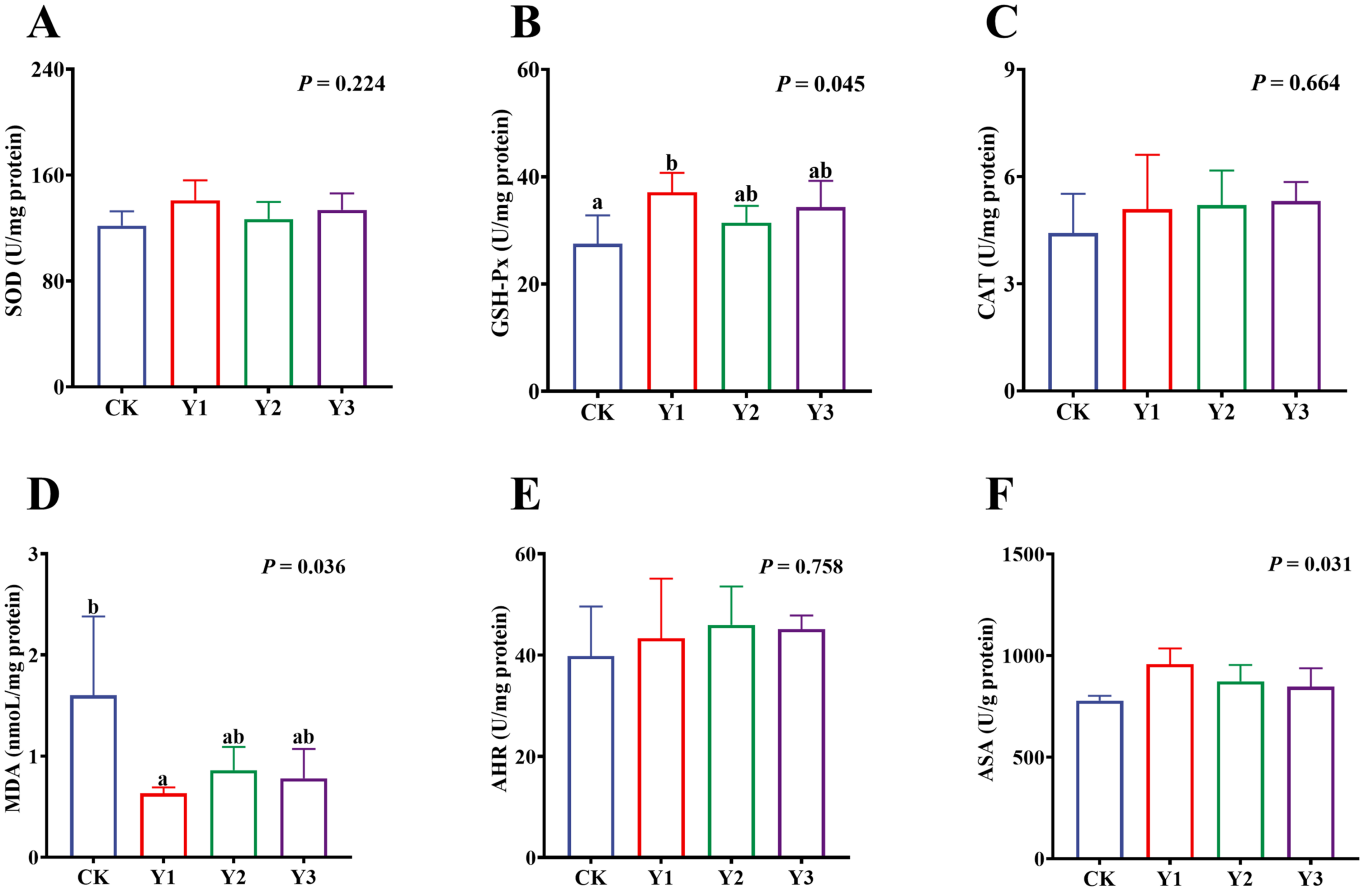
Effect of AOE on antioxidant function in the jejunal mucosa of ducks. SOD: Superoxide Dismutase; GSH-Px: Glutathione Peroxidase; CAT: Catalase; MDA: Malondialdehyde; AHR: Anti-hydroxyl radical; ASA: Anti-superoxide anion. ^a,b^ Bars with different superscripts indicate a significant difference (*n* = 6, *p* < 0.05).

**Figure 7:**
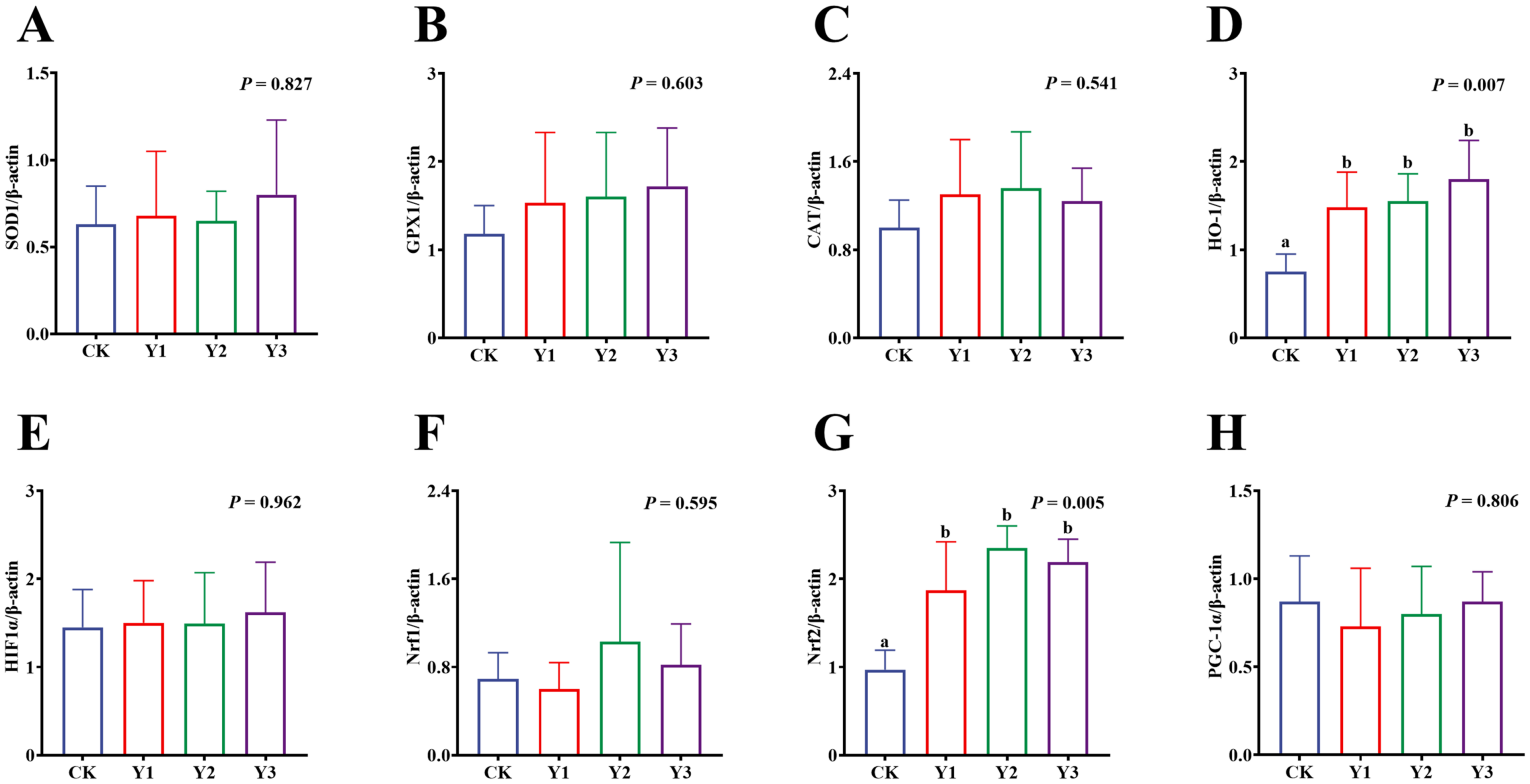
Effect of AOE on mRNA relative expression of antioxidant function-related genes in the jejunal mucosa of ducks. β-actin: beta-actin; SOD1: superoxide dismutase 1; GPX1: glutathione peroxidase 1; CAT: catalase; HO-1: heme oxygenase 1; HIF1A: hypoxia inducible factor 1 subunit alpha; Nrf1: nuclear factor erythroid 1-related factor 1; Nrf2: nuclear factor erythroid 2-related factor 2; PGC1α: peroxisome proliferator-activated receptor gamma (PPAR-G) coactivator 1 alpha. ^a,b^ Bars with different superscripts indicate a significant difference (*n* = 6, *p* < 0.05).

Compared to the CK group, the sucrase activity in the Y2 group was significantly (*p* < 0.05) enhanced (Figure 5C). In contrast, the maltase and lactase activities showed no observed significant (*p* > 0.05) differences in among the groups (Figures 5A,B). The GSH-Px and ASA levels in the Y1 group were significantly (*p* < 0.05) increased, and the MDA content was significantly (*p* < 0.05) reduced (Figures 6C,D,F). In contrast, the SOD, GSH-Px and AHR activities in these groups remained unchanged (*p* > 0.05) (Figures 6A,B,E). Furthermore, the mRNA expression of *HO-1* and *Nrf2* in the AOE groups (Y1, Y2, and Y3) were significantly (*p* = 0.007, *p* = 0.005) up-regulated (Figures 7D,G). In contrast, although the expression of *SOD1*, *GPX1*, *CAT*, *HIF1α*, *Nrf1*, and *PGC-1α* were regulated but there was no difference (*p* > 0.05) (Figures 7A–C,E,F,H).

### 3.4 Effects of the dominant microbial genus on the jejunal indicators oxidation and immune

**Figure 8:**
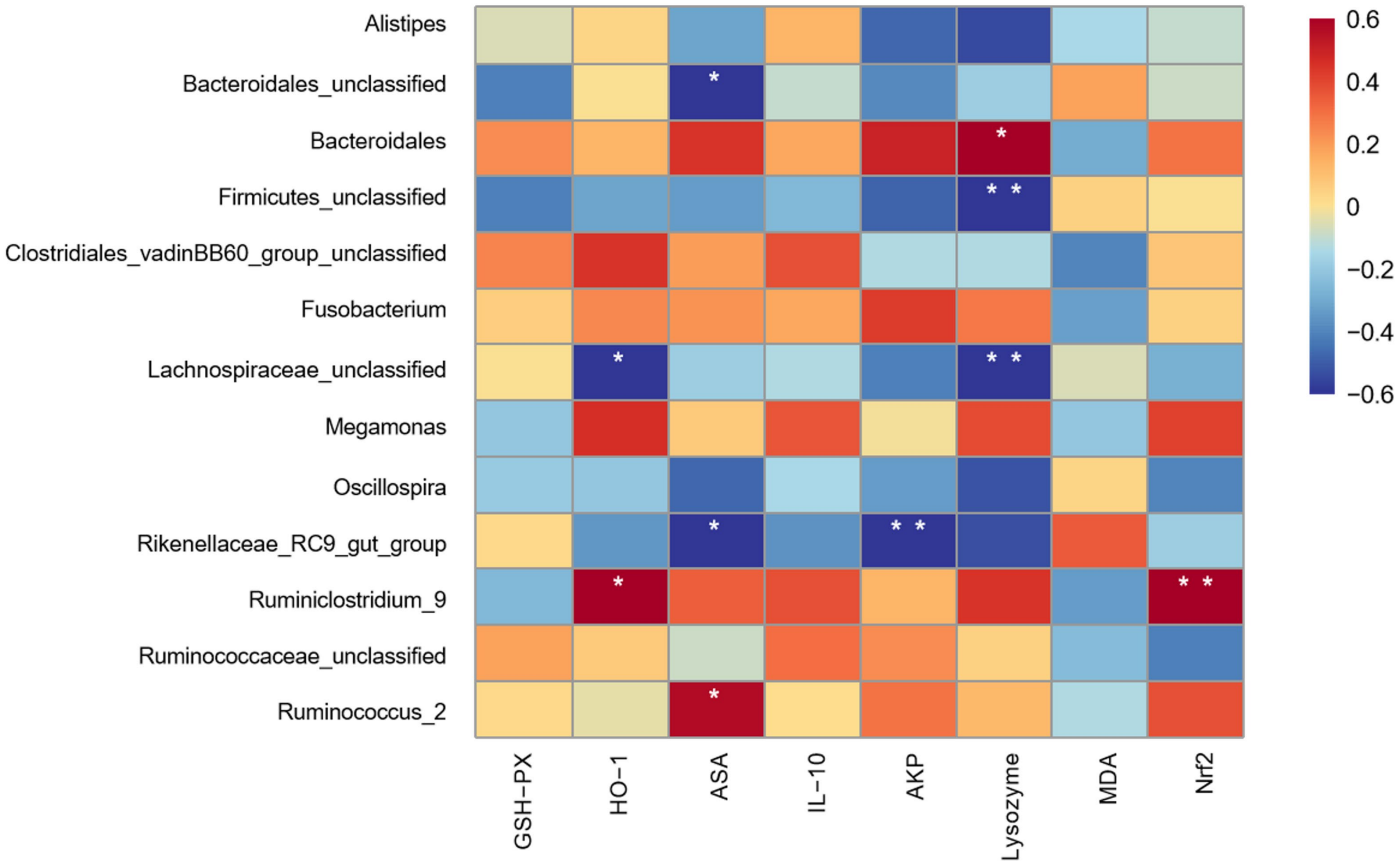
Spearman’s correlation analysis of dominant bacterial genera with oxidative immune indicators of jejunal mucosa. GPH-PX: Glutathione Peroxidase; HO-1: heme oxygenase 1; ASA: Anti-superoxide anion; IL-10: interleukin-10; AKP: Alkaline phosphatase; MDA: malondialdehyde; Nrf2: nuclear factor erythroid 2-related factor 2. Colours ranging from blue to red indicate a shift from negative to positive correlation. Correlation coefficients marked with * denote statistically significant differences (*p* < 0.05), while those marked with ** indicate highly significant differences (*p* < 0.01) (*n* = 9).

The spearman correlation analysis is presented in Figure 8. The results showed that the function of oxidation and immunity in jejunum exhibited a certain degree of correlation with the microbial genus in caecum. The *HO-1* gene expression was positively correlated (*p* < 0.05) with *Ruminiclostridium_9* and negatively correlated (*p* < 0.05) with *Lachnospiraceae_unclassified*. The ASA activity was positively correlated (*p* < 0.05) with *Ruminococcus_2* and negatively correlated (*p* < 0.05) with *Rikenellaceae_RC9_gut_group* and *Bacteroidales_unclassified*. The AKP activity was positively correlated (*p* < 0.01) with *Rikenellaceae_RC9_gut_group*. Moreover, the lysozyme activity was positively correlated (*p* < 0.05) with *Bacteroidales* and negatively correlated (*p* < 0.01) with *Firmicutes_unclassified* and *Lachnospiraceae_unclassified.* The *Nrf2* gene expression was positively correlated (*p* < 0.01) with *Ruminiclostridium_9*.

### 3.5 Effect of AOE on SCFA in cecal contents

As shown in Table 2, the acetic acid and isobutyric acid in the Y2 group were significantly (*p* < 0.05) increased, indicating that the addition of AOE would exert a notable effect on gut microbial metabolites.

**Table 2:** Effect of AOE on the cecal contents of ducks.

Items	CK	Y1	Y2	Y3	*p* value
Acetic acid, mg/g	5.48 ± 1.53^b^	6.82 ± 1.62^ab^	8.92 ± 0.44^a^	7.63 ± 1.31^ab^	0.040
Propionic acid, mg/g	1.45 ± 0.41	2.15 ± 0.12	1.78 ± 0.25	1.86 ± 0.45	0.142
Butyric acid, mg/g	1.18 ± 0.30	1.29 ± 0.36	1.34 ± 0.29	1.14 ± 0.22	0.761
Isobutyric acid, mg/g	2.50 ± 0.11^b^	2.87 ± 0.42^ab^	3.40 ± 0.27^a^	3.01 ± 0.37^ab^	0.025
Valeric acid, mg/g	0.36 ± 0.11	0.43 ± 0.10	0.39 ± 0.03	0.40 ± 0.06	0.742
Isovaleric acid, mg/g	6.67 ± 1.99	5.89 ± 0.23	5.46 ± 0.75	6.41 ± 0.79	0.471

^*^Within a row, means without a common superscript differ at *P* < 0.05.

## 4 Discussion

Herbs and their extracts are known for wide availability, environmental sustainability, anti-inflammatory and antioxidant properties (18, 19). They are prepared as additives into feed to enhance animals intestinal health, which can promote improve intestinal morphology, repair intestinal barriers, enhance antioxidant capacity, strengthen tight junction proteins, and alleviate heat stress in ducks (20–22). This study revealed that *A. oxyphylla* stems and leaves were rich in fatty acyls, isoprenoid lipids, flavonoids, hydroxy acids and their derivatives. These results showed some differences in composition compared to the results of the previous study by Ying et al. (8), who found that the principal constituents in the *A. oxyphylla* stems and leaves are flavonoids, phenolic acids, and terpenoids. The observed discrepancy in composition may be attributable to variations in detection methodologies and sample sources. Nevertheless, key bioactive components were consistently identified across studies, including fatty acyls, terpenoids, flavonoids, and phenols. Thapa et al. (23) reported that the sesquiterpenoids isolated from *A. oxyphylla* fruit extracts exhibited dose-dependent antioxidative effects, which could revers tert-butyl hydroperoxide-induced oxidative stress in adipose-derived mesenchymal stem cells. Notably, isoprenoid lipids and sesquiterpenoids represent the significant components of terpenoids, which are widely known for neuroprotective, anti-inflammatory, and antioxidant properties (24–26). The results suggest that the isoprenoid lipids in the *A. oxyphylla* stems and leaves may possess analogous biological functions similarly. Furthermore, the flavonoids in the *A. oxyphylla* stems and leaves were found to be rich. The compounds possess various advantages, including immunomodulatory, anti-inflammatory, and antioxidant activities. Tu et al. (27) demonstrated that dietary supplementation with 500 mg/kg of *Lycium barbarum* flavonoids in 1-day-old Cherry Valley ducks, which could significantly up-regulate mRNA expression of antioxidant-related genes (*SOD1*, *CAT*) and down-regulate pro-inflammatory factors (Bcl-2-associated X, *TNF-α*). In conclusion, the isoprenoid lipids (terpenoids) and flavonoids in the extracts are key components to improve meat quality, blood lipid metabolism and intestinal health in Jiaji ducks. However, further research is required to identify the more specific components within the substances that play a key role.

The intestinal mucosa is responsible for maintaining intestinal homeostasis through its chemical barrier and immunomodulatory functions (28). Furthermore, the secretion of active enzymes is imperative for suppressing intestinal inflammatory responses (29, 30). Compared with the CK group, the activities of lysozyme and AKP in the Y1 and Y2 groups were exhibited higher levels. Lysozyme, a pivotal constituent of the intestinal chemical barrier, exerts a direct bactericidal effect on pathogenic bacteria by hydrolyzing the peptidoglycan component of bacterial cell walls, which can enhance antimicrobial defense and reduce bacterial colonization (31). Similarly, AKP blocks binding to Toll-like receptor 4 on intestinal epithelial cells by dephosphorylating lipopolysaccharides from gram-negative bacteria (32). Consequently, the both enzymes can enhance the intestinal immunity and chemical barrier function to reduce pathogenic bacteria growth, thereby maintaining the health of Jiaji ducks. Moreover, the mRNA expression of *IL-10* was significantly up-regulated in the AOE groups (Y1, Y2, and Y3) compared to the CK group. *IL-10* has been shown to regulate pro-inflammatory responses by blocking *NF-κB*, supporting B-cell growth, and increasing immunoglobulin levels (33, 34). It is worth noting that despite its documented anti-inflammatory properties in other contexts (4), no significant disparities (except *IL-10*) were identified in the mRNA expression of other inflammation-related factors and tight junction proteins in this study. This could be attributed to the healthy function of AOE, which mitigated severe inflammatory reactions in the fattening Jiaji ducks, reducing the need for excessive regulatory responses to maintain homeostasis. Therefore, the extracts may was effective for immune challenge or chronic inflammation, but sub-threshold in healthy Jiaji ducks for immunomodulation. Further investigation in immune challenge or chronic inflammation is necessary to clarify the anti-inflammatory mechanisms of AOE.

Oxidative stress is a pathological condition arising from an imbalance in the body’s oxidative homeostasis (35). The condition is characterized by excessive accumulation of free radicals and reactive oxygen species, leading to intestinal cell damage (36). During periods of high-density breeding, Jiaji ducks exhibit a predisposition to endogenous reactive oxygen overload. The state of oxidative imbalance probably due to environmental pollutants, high-energy diets, and intensive management practices. It is important to note that an imbalance in oxidation can result in several adverse effects, including intestinal inflammation, impairing intestinal barrier function, microbiota-immune dysregulation, reducing growth performance, and increasing disease susceptibility (37). In this study, the GSH-Px and ASA activities in the Y1 group were significantly increased, and the MDA content was significantly reduced. GSH-Px plays a pivotal role in cellular protection through its function as the catalyst for hydrogen peroxide reduction via reduced glutathione (38). MDA, a lipid peroxidation product, has been demonstrated to directly correlate with the severity of oxidative damage (39). The results indicated that 30 mg/kg AOE could maintain intestinal redox homeostasis through enhancing antioxidant enzyme activity and inhibiting lipid peroxidation. Similarly, Qiu et al. (40) reported that the sesquiterpenoids from *A. oxyphylla* fruits were able to reduce MDA levels in cells subjected to oxidative stress. Furthermore, the mRNA expression levels of *Nrf2* and *HO-1* in the AOE groups (Y1, Y2, and Y3) were up-regulated. As a master regulatory pathway of antioxidant responses, *Nrf2* can activate downstream genes (e.g., *HO-1*) by binding to antioxidant response elements, thereby enhancing cellular antioxidant capacity (41–43). For instance, Kong et al. (44) demonstrated that dietary glycerol monolaurate alleviated jejunal oxidative stress in broilers via *Nrf2/HO-1* pathway activation. Bian et al. (45) found that the Oxyphylla A extracts from *A. oxyphylla* enhanced the antioxidant enzyme activities in mice by modulating the *Nrf2-Keap1-HO-1* axis. These results are similar to our findings, indicating that the AOE may synergistically activate the *Nrf2/HO-1* signaling pathway to maintain intestinal redox balance in Jiaji ducks. Nevertheless, further studies are required in order to elucidate the underlying mechanisms.

The complex microbial flora in cecum plays a crucial role in maintaining intestinal homeostasis and the host’s health status. The alterations of microbial composition in cecum can significantly impact the oxidation and immunity in gut, and metabolites as well as other related microbial indicators (46). The Spearman correlation analysis were performed to investigate the correlation between the function of oxidation and immunity in jejunal mucosa with the abundance of microbial genera levels in cecum. The results showed that *Lachnospiraceae_ unclassified* was negatively correlated with *HO-1* and *Lysozyme* activity. *Rikenellaceae_RC9_gut_group* was negatively correlated with ASA and AKP. *Ruminiclostridium_9* was positively correlated with *HO-1* and *Nrf2*. *Ruminococcus_2* was positively correlated with ASA activity. Some strains of *Ruminiclostridium_9* are involved in the fermentation of dietary fiber to produce SCFA, and these SCFA can activate the *Nrf2* signaling pathway and up-regulate the downstream target gene *HO-1* (47, 48). These strains may benefit by activating the host antioxidant defense pathway, while the up-regulation of *Nrf2* and *HO-1* in the AOE groups (Y1, Y2, and Y3) supported the findings. *Lachnospiraceae_unclassified* belongs to the *Lachnospiraceae* family and may be associated with enteric and extraintestinal diseases (49). The strains of *Lachnospiraceae_unclassified* colonization and invasion were reduced when lysozyme activity was increased, indicating that the activation of basal immunosurveillance and defense mechanisms in the jejunum of Jiaji ducks. The above study found a relationship between the AOE supplementary in cecal microbial composition with the regulation of oxidation and immunity in jejunum, which could maintain oxidation and immunity homeostasis in the gut of Jiaji ducks. Additionally, SCFA is a key the metabolite of cecum microbial fermentation. It alleviates oxidative stress, inhibits inflammatory responses, and mediates microbes’ regulation to gut metabolism and immune functions (50). In this study, the levels of acetic acid and isobutyric acid were significantly increased in the Y2 group. Similarly, Gebeyew et al. (51) found that *Ruminococcaceae* can use dietary fiber from the cecum to produce SCFA, which promotes the production of acetic acid and isobutyric acid. Acetic acid can be a substrate for fatty acid and cholesterol synthesis, which can enhance ileal motility by affecting ileal contractions (52). The results suggested that the SCFA content in the cecum of Jiaji ducks could be changed by the AOE supplementary, thereby altering the energy supply to the gut and indirectly influencing the function of oxidation and immunity. In conclusion, adding appropriate amounts of AOE to the diets of Jiajj ducks can alter the composition of the intestinal flora, and indirectly modulating the function of oxidation and immune through changing SCFA.

## 5 Conclusion

This study aimed to investigate the effects of AOE on the intestinal health of Jiaji ducks. The principal findings demonstrate that the *A. oxyphylla* stems and leaves constitute a natural source of anti-inflammatory and antioxidant agents. Importantly, this study also found that the optimal dosage for maintaining intestinal health in Jiaji ducks was determined to be 30 mg/kg. These findings indicate that the extracts from *A. oxyphylla* stems and leaves can enable more efficient utilization of *A. oxyphylla* resources and hold potential for application in maintaining poultry gut health. While this study investigated the effects of AOE on healthy Jiaji ducks during the fattening phase, further investigations in its therapeutic efficacy during immune challenges or chronic inflammation are necessary. Moreover, future studies should also focus on optimizing AOE extraction processes to reduce costs and increase extraction rates, thereby enabling its widespread application in poultry production. This study provides a strategy for utilizing *A. oxyphylla* by-products and holds significant potential for promoting the sustainable development of the Jiaji duck industry.

## Data Availability

The original contributions presented in the study are included in the article/Supplementary material, further inquiries can be directed to the corresponding authors.
