# Supplementary material for: Effects of Alpinia oxyphylla stems and leaves extracts on immune function, antioxidant function, and microbial flora composition in the intestinal tract of Jiaji ducks
Source: Front Vet Sci. 2025 Nov 27;12:1662049. doi: 10.3389/fvets.2025.1662049 (PMC12696864; doi:10.3389/fvets.2025.1662049)
Supplement: Supplementary file 1 [file Table_1.DOCX]

Table S1 Composition and nutrition levels of the basal diet.

| Ingredients | Content (%) | Nutrient Levels | Content (%) |
| --- | --- | --- | --- |
| Corn | 70.53 | Me (MJ/kg) | 12.27 |
| Soybean meal | 23.5 | Crude protein | 16.79 |
| Wheat bran | 1.7 | Crude fat | 3.05 |
| CaHPO_4_ | 1.37 | Crude fiber | 2.63 |
| Limestone | 0.9 | Calcium | 0.46 |
| L-Lysine | 0.14 | Available P | 0.35 |
| DL-Methionine | 0.18 | L-Lysine | 0.95 |
| Premix^1^ | 1.68 | Methionine | 0.45 |
| Total | 100 |  |  |

^a^One kilogram of multiple vitamin premix contained: vitamin A, 50,000,000 IU; vitamin B1, 10,000 mg; vitamin B2, 20,000 mg; vitamin B6, 10,000 mg; vitamin B12, 5,000 mg; vitamin C, 4,000 mg; vitamin D, 1,000,000 IU; vitamin E, 60,000 IU; vitamin K3, 8,000 mg; folic acid, 2,500 mg; niacin, 80,000 mg; pantothenic acid, 30,000 mg; biotin, 2,200 mg; Cu, 5 g; Fe, 50 g; Zn, 55 g; Mn, 55 g; I, 0.3 g; Se, 0.22 g.
